# The diagnosis and treatment of disorders of nucleic acid/nucleotide metabolism associated with epilepsy

**DOI:** 10.1186/s42494-025-00201-x

**Published:** 2025-04-01

**Authors:** Yuqing Shi, Zihan Wei, Yan Feng, Yajing Gan, Guoyan Li, Yanchun Deng

**Affiliations:** 1 Xi’an Medical University, Xi’an, 710021 People’s Republic of China; 2 Department of Neurology, Xijing Hospital, Fourth Military Medical University, 127 West Changle Road, Xi’an, 710032 People’s Republic of China; 3 Xijing Institute of Epileptic Encephalopathy, Shaanxi, Xi’an, 710065 People’s Republic of China

**Keywords:** Nucleic acid/nucleotide metabolism, Epilepsy, Gene genetic variants

## Abstract

Epilepsy is a prevalent paroxysmal disorder in the field of neurology. Among the six etiologies of epilepsy, metabolic causes are relatively uncommon in clinical practice. Metabolic disorders encompass amino acid metabolism disorders, organic acid metabolism disorders, and other related conditions. Seizures resulting from nucleic acid/nucleotide metabolism disorders are even more infrequent. This review provides an overview of several studies on nucleic acid/nucleotide metabolism disorders associated with epilepsy, including adenosine succinate lyase deficiency, Lesch-Nyhan syndrome, and aminoimidazole carboxamide ribonucleotide transformylase/inosine monophosphate cyclohydrolase (ATIC) deficiency, among others. The potential pathogenesis, phenotypic features, diagnostic pathways, and therapeutic approaches of these diseases are discussed in this review. The goal is to help clinicians make an accurate diagnosis when encountering rare nucleic acid/nucleotide metabolism disorders with multi-system symptoms and manifestations of epilepsy.

## Background

Nucleic acids play a crucial role in carrying genetic information and determining the genotype of cells and individuals. Purine and pyrimidine nucleotides are vital constituents of DNA and RNA, serving as essential building blocks for numerous biological processes. Pyrimidines are involved in phospholipid and glycogen synthesis, as well as sialylation and glycosylation of proteins, while purines act as intracellular and intercellular signaling molecules, including guanine-related G protein-coupled receptors. Additionally, ATP serves as the primary energy source for various biological processes [1]. Clinical manifestations commonly associated with disorders of nucleic acid or nucleotide metabolism encompass psychomotor retardation, seizures, microcephaly, among others [2–4]. Diagnosis can be established through specific manifestations observed during auxiliary examinations, enzyme tests, and genetic testing. Such syndromes are characterized by clinical manifestations of the nervous system. The common symptoms include psychomotor retardation, seizures, microcephaly, etc. In laboratory testing, a modified Bratton-Marshall, high-performance liquid chromatography (HPLC), ultraviolet detection, or mass spectrometry can be used to determine precursor substances associated with enzyme deletion or to determine enzyme activity in cellular tissues such as red blood cells and fibroblasts. Treatment primarily focuses on symptomatic management; however, targeted interventions addressing the underlying pathogenic mechanisms and metabolic disorders may also be employed. This article provides an overview of syndromes related to nucleic acid/nucleotide metabolism disorders accompanied by epilepsy symptoms. It summarizes their etiology, clinical features, and treatment options while reviewing pertinent literature to aid clinicians in accurately diagnosing rare cases presenting with multi-system symptoms associated with nucleic acid/nucleotide metabolism disorders.

## Disorders of nucleic acid/nucleotide metabolism

### Adenosine succinate lyase deficiency

**Fig. 1:**
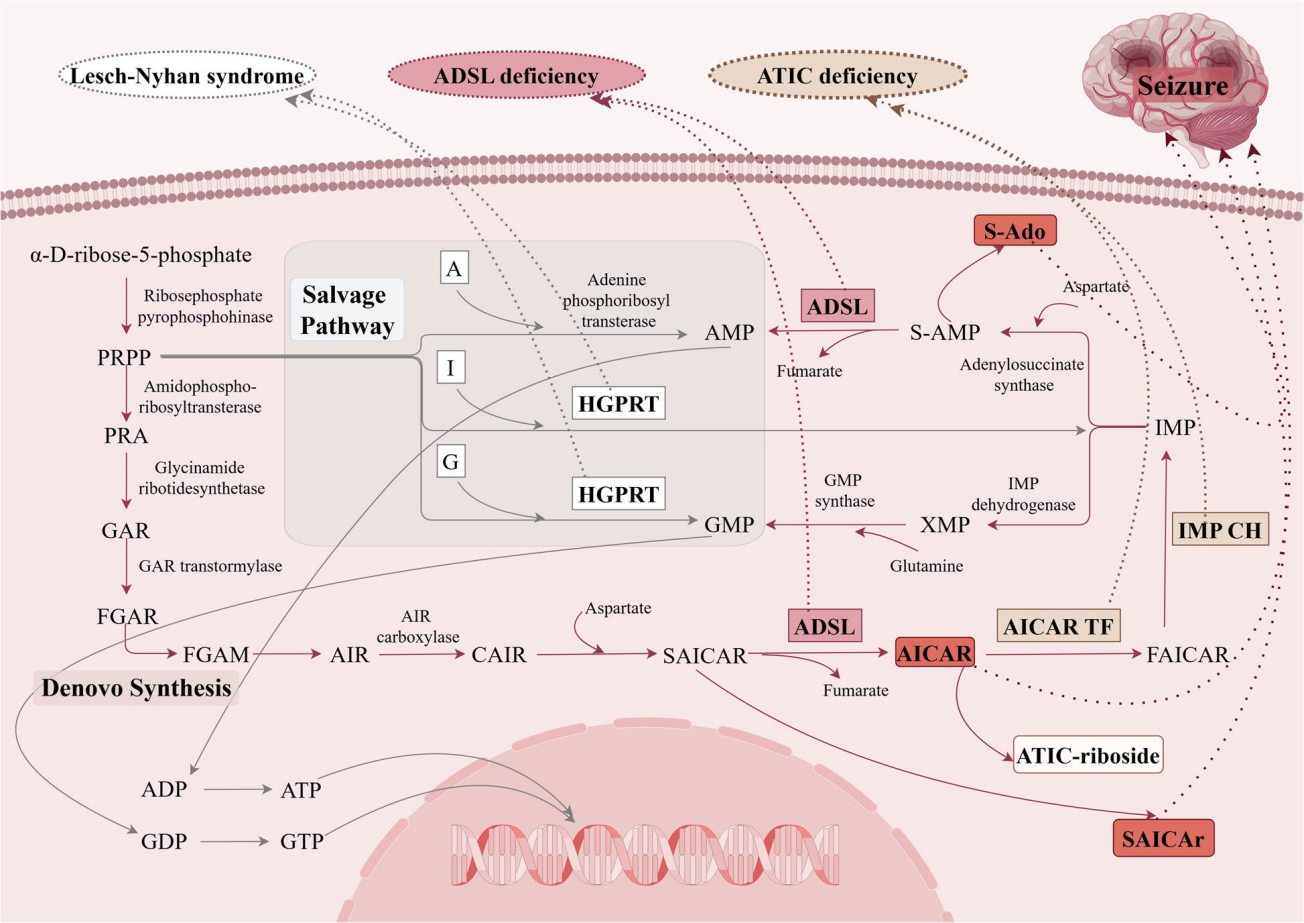
Schematic diagram of purine nucleotide anabolism. Red boxes in bold indicate substances that produce toxic effects on the nervous system. Abbreviations: A, Adenosine; ADSL, Adenylosuccinate lyase; AIR, Aminoimidazole ribotide; AICAR, Aminoimidazolecarboxamide ribose; AICAR TF, Amino imidazole carboxamide ribosidetransformylase; AMP, Adenosine monophosphate; ADP, Adenosine diphosphate; ATP, Adenosine triphosphate; ATIC, Amino imidazole carboxamide ribosidetransformylase/I inosine monophosphorus cyclohydrolase; CAIR, Carboxyl amino imidazoleribotide; FAICAR, Formylaminoimidazole-carbamide ribose; FGAM, Formyl glycin amidine ribotide; FGAR, Formyl glycin amide ribotide; G, Guanine; GAR, Glycinamide ribotide; GMP, Guanine monophosphate; GDP, Guanine diphosphate; GTP, Guanine triphosphate; HGPRT, Hypoxanthine–guanine phosphoribosyl transferase; I, Inosine; IMP, Inosine monophosphate; IMP CH, Inosine mono phosphorus cyclohydrolase; PRPP, Phosphoribosyl pyrophosphate; PRA, Phosphoribosyl amine; SAICAR, Succinylaminoimidazolecarboxamide ribose; S-AMP, Adenylosuccinate; XMP, Xanthosine monophosphate

Adenosine succinate lyase (ADSL) deficiency is a rare autosomal recessive disorder of purine nucleotide metabolism. The disease-causing gene is *ADSL*, located at 22q13.1, encoding ADSL. The main function of ADSL is to participate in the conversion of succinylaminoimidazolecarboxamide ribose (SAICAR) to aminoimidazolecarboxamide ribose (AICAR) in the de novo purine synthesis pathway and the conversion of inosine monophosphate (IMP) to adenosine monophosphate (AMP) through adenylosuccinate (S-AMP) in the purine nucleotide cycle (Fig. 1). Variants in the *ADSL* gene can lead to ADSL deficiency, which blocks the above pathway, that is, the de novo synthesis of adenine and guanine nucleotides is reduced, and it has an impact on tissues with high energy demand, such as brain tissue. The deficiency of ADSL can also lead to the accumulation of SAICAR and S-AMP in the form of dephosphorylation, that is, SAICAr and S-Ado, two toxic intermediate metabolites, leading to neurotoxicity [5, 6]. In addition, some studies have established zebrafish models that mimic human ADSL deficiency (National Natural Science Foundation of China, Project Number: 81371264), the developmental defects of monoamine neurons and gabaergic neurons in ADSL deficient embryos were found by transgenic marker fish lines, which were associated with the lack of AMP development at early embryonic stage, leading to neurological symptoms such as epilepsy.

The common clinical manifestations of ADSL deficiency include psychomotor retardation, hypotonia, seizures, and behavioral changes. The seizure types of ADSL deficiency include myoclonic seizures, focal seizures, generalized tonic–clonic seizures, status epilepticus, infantile spasms, etc. The severity of the disease can be determined by measuring the ratio of S-Ado/SAICAr in cerebrospinal fluid. The smaller the ratio of S-Ado/SAICAr in cerebrospinal fluid, the higher the severity of the disease [7]. Clinically, it can be divided into the following three types according to the severity of symptoms [2]. The first is the neonatal lethal type, whose clinical manifestations include pulmonary hypoplasia, severe and fatal neonatal encephalopathy, respiratory failure, and intractable seizures. Early death may occur, and the ratio of S-Ado/SAICAr in cerebrospinal fluid is less than 1 [8]. The second type, called type I ADSL deficiency (severe type), is characterized by severe psychomotor disorders, developmental delay, early seizures, coma, etc. In the first few weeks of life, and the ratio of S-Ado/SAICAr in cerebrospinal fluid is approximately equal to 1 [9–12]. The last is type II ADSL deficiency (moderate/mild), characterized by mild to moderate psychomotor retardation, hypotonia, and seizures several years after birth, with a ratio of S-Ado/SAICAr in cerebrospinal fluid ≥ 2 [7, 9, 12–14].

The clinical diagnosis of ADSL deficiency can be determined by the modified Bratton-Marshall test [15], HPLC with ultraviolet detection [16], and using HPLC combined with electrospray ionization (ESI) tandem mass spectrometry (MS/MS) to develop a high-throughput urine screening technology [17]. The concentrations of SAICAr and S-Ado in urine and cerebrospinal fluid were significantly increased, and the concentrations of SAICAR and S-Ado in blood were also increased correspondingly. The ratio of S-Ado/SAICAr in cerebrospinal fluid may help to diagnose the severity of the disease. Brain atrophy, decreased myelination, thinning of the corpus callosum, white matter abnormalities, and cerebellar hypoplasia can be detected by magnetic resonance imaging (MRI) [2, 18–21]. Electroencephalogram (EEG) may show extensive spikes and waves, burst-suppression manifestations, etc [2].

There is no specific treatment for ADSL deficiency currently. It has been reported that the use of D-ribose and uridine [22, 23] can improve the motor coordination of patients and control seizures, but there is no more effective treatment data to support this. S-adenosine-l-methionine has been reported to be used in the treatment of ADSL deficiency, but the efficacy is not good [17]. The treatment of antiseizure medications is related to the type of seizures. Clinically, most patients with ADSL deficiency are treated with multi-medication combination therapy, and the frequency of drug resistance is high. A ketogenic diet is an effective way to treat refractory epilepsy at present [24, 25]. The ketogenic diet can produce hypoglycemia and decrease PH value, increase adenosine and ATP levels, and increase ketone bodies, that is, an increase of acetone, can have an anticonvulsant effect [26] which can also increase the inhibitory neurotransmitter γ-aminobutyric acid (GABA) against epilepsy. When ketogenic diet therapy is applied, patients should be comprehensively and systematically examined before medication, to better prevent the influence of side effects on patients.

### Lesch-Nyhan syndrome

Lesch-Nyhan syndrome (LNS) is an X-linked recessive disorder of purine nucleotide metabolism caused by variants in the gene encoding hypoxanthine–guanine phosphoribosyl transferase (HGPRT). The *HPRT* gene is located at Xq26.2–Xq26.3, with a total length of about 44 kb and 9 exons [27]. The gene encodes HGPRT, which catalyzes the conversion of inoxanine to inosine monophosphate (IMP) and guanine to guanine monophosphate (GMP) (Fig. 1). Variants in the *HPRT* gene lead to the deficiency of HGPRT, resulting in the accumulation of phosphoribosyl pyrophosphate (PRPP), which in turn accelerates the de novo synthesis of purine [28, 29], and causes guanine and inosine to be metabolized to uric acid [30, 31]. The brain relies on the salvage pathway of purines, so the production of guanine and adenine nucleotides in brain tissue is reduced.

When HGPRT is completely deficient, it is called Lesch-Nyhan syndrome. Most of the patients are male. The common clinical manifestations of this disease include psychomotor retardation, intellectual disability, spastic cerebral palsy, choreoathetosis, urinary stones, self-destructive bite, and seizures [3]. The increase of uric acid can be present from birth, the deposition of uric acid crystals can lead to urinary calculi, orange crystals can be left in the diaper of children in infancy, and delayed treatment may lead to renal failure. Hypotonia and psychomotor retardation may occur in the first year of life, other symptoms such as choreiform athetosis may manifest in the first few years of life, and self-destructive bites of the fingers and lips usually appear between the ages of 2 and 4 years [32]. Patients with LNS may present with seizures, but because more patients with LNS may present with dyskinesia, attention should be paid to identifying other clinical manifestations such as spasms as seizures. Christy et al. reported a case of LNS patient with subclinical status epilepticus with respiratory failure and severe respiratory acidosis in 2016. In this report, it was suggested that the respiratory failure and sudden death tendency of LNS patients may be related to seizures [33]. The neurological manifestations of LNS are related to the dysfunction of the dopaminergic neurotransmitter system in the basal ganglia. A significant increase in hypoxanthine can be observed in the biochemical examination of LNS patients, which can produce toxic effects on the nervous system by changing adenosine transport [34, 35] and affecting Na^+^-K^+^-atpase activity [36, 37].

Laboratory examination of LNS may suggest hyperuricemia, but LNS cannot be completely excluded when the uric acid level is not elevated on clinical examination. The diagnosis of LNS can also be made by measuring the activity of HGPRT in peripheral red blood cells or skin fibroblasts and detecting *HPRT* gene variants. The EEG of patients with this disease shows nonspecific slowing or disorder, and their neuroimaging studies can show brain atrophy with reduced brain volume and caudate nucleus volume.

The treatment of hyperuricemia is one of the key points in the treatment of LNS. Allopurinol, as a xanthine oxidase inhibitor, can be used for the treatment of hyperuricemia [38, 39]. The self-injurious behavior of LNS patients can be improved by physical restraint management, such as lip shields and tooth extraction [40]. In the treatment of neurological symptoms of LNS, such as improving dystonia and behavioral symptoms, baclofen can be used as a possible functional dopamine antagonist and its anti-anxiety effect, and the improvement effect is better [31, 41]. Gabapentin as an antiseizure medication can also be used in the treatment of LNS. It has been reported that gabapentin is partially effective in the control of dystonia and self-injury behavior [42]. The combination of S-adenosine-L-methionine (SAMe) and risperidone is also a feasible treatment to improve the neurological symptoms of LNS. SAMe is a major methyl donor, which affects the central nervous system function through the cellular transmethylation pathway. SAMe also has a significant antidepressant effect and can supplement the purine pool as an adenosine donor [43]. Baclofen or benzodiazepines can improve spasticity and clonic seizures in LNS. At present, a variety of treatments for LNS are being studied, such as deep brain stimulation [44], bone marrow transplantation [45] and carbamazepine [46].

### ATIC deficiency

Amino imidazole carboxamide ribosidetransformylase/I inosine monophosphorus cyclohydrolase (ATIC) deficiency is a rare autosomal recessive disorder of purine nucleotide metabolism. So far, few cases have been reported. The disease is associated with variants in the *ATIC* gene, located on chromosome 2q35, which encodes a bifunctional enzyme [47] involved in the de novo purine synthesis pathway, namely amino imidazole carboxamide ribosidetransformylase (AICAR TF) and inosine monophosphorus cyclohydrolase (IMP CH). The former converts AICAR to formylaminoimidazole-carbamide ribose (FAICAR), and the latter converts FAICAR to IMP (Fig. 1). When the *ATIC* gene is varied, the effect on AICAR TF is greater than that on IMP CH, resulting in the accumulation of AICAR and its dephosphorylation product ATIC-riboside. Studies have shown that ATIC-riboside has a significant inhibitory effect on carbohydrate [48] and liver lipid metabolism [49], and can stimulate glucose uptake. AICAR may have neurotoxic effects on the developing brain [50], and AICAR is an activator of AMP-activated protein kinase (AMPK) [51, 52].

These mechanisms may be related to a series of clinical manifestations of ATIC deficiency, including neurodevelopmental disorders, severe visual impairment such as chorioretinal atrophy, prenatal growth disorders, severe scoliosis, facial deformities, and early-onset epilepsy. Rare manifestations such as aortic coarctation, chronic hepatocytes, mild genital malformations, and nephrocalcinosis may also occur [53]. The seizure types of ATIC may include epileptic spasms, generalized tonic seizures, focal seizures with disturbance of consciousness, etc [53, 54]. Most of them are drug-resistant epilepsy. The pathogenesis of ATIC deficiency may be related to the toxic effects of accumulated intermediate metabolites, or the deficiency of purine synthesis due to the block of de novo purine synthesis pathway [55]. However, during embryonic development and organogenesis, more purine-dependent de novo synthesis pathways are used up [54]. In 2020, Ramond et al. reported three cases of ATIC deficiency and described the status of the first case of ATIC deficiency [53]. All three new ATIC deficiency patients presented with late intrauterine growth retardation (IUGR).

In the biochemical examination of ATIC deficiency, the levels of ATIC ribosides in urine and cerebrospinal fluid are significantly increased, and AICAR and its derivatives in erythrocytes and fibroblasts are increased, which may be related to adenosine kinase phosphorylating the uptake of ATIC ribosides to AICAR. It may also be explained that the decrease in ATP concentration in erythrocytes is due to the conversion of ATP into AMP by this phosphorylation process. In addition, S-Ado and SAICARr can also be increased in this disease, which is related to the inverse inhibition of ADSL after accumulation of AICAR. The absence of purine is not found in ATIC deficiency, which may be related to the compensatory synthesis of purine by the salvage synthesis pathway. Enzyme examination showed that the AICAR TF activity in the fibroblasts was severely deficient, and the residual IMP CH activity was 40% of the normal value. MRI showed abnormal signals in the dorsal nucleus of the brainstem, thickened corpus callosum, and delayed myelination.

There is no specific treatment for ATIC deficiency, and the cytotoxic effect of accumulated intermediate metabolites may be the pathological mechanism of this disease [55]. Therefore, blocking the cytotoxic effect may be an effective target for treatment. In addition, antiseizure medications can be used for the epileptic symptoms of this disease, but the clinical heterogeneity is high. Different patients have different responses to antiseizure medications, and individualized effective medication is the key. In the case reported by Ramond et al., epilepsy is mostly characterized by drug resistance [53].

### Adenosine monophosphate deaminase deficiency

**Fig. 2:**
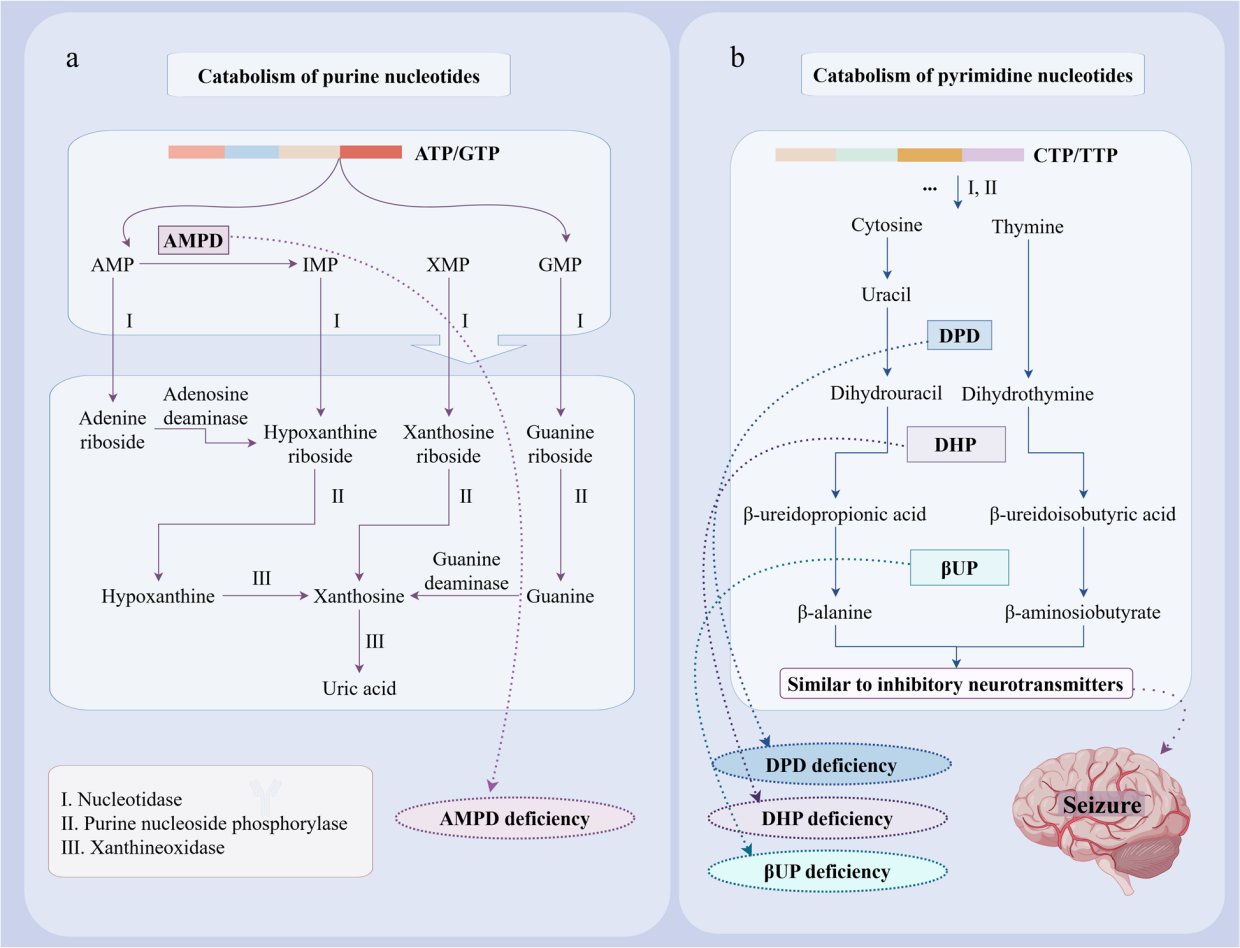
Schematic diagram of purine and pyrimidine nucleotide catabolism. Abbreviations: AMP, Adenosine monophosphate; AMPD, Adenosine monophosphate deaminase; ATP, Adenosine triphosphate; CTP, Cytidine triphosphate; DHP, Dihydropyrimidinase; DPD, Dihydropyrimidine dehydrogenase; GMP, Guanine monophosphate; GTP, Guanine triphosphate; IMP, Inosine monophosphate; TTP, Thymidine triphosphate; XMP, Xanthosine monophosphate; βUP, β-ureidopropionase

The *AMPD2* gene encodes adenosine monophosphate deaminase (AMPD), an enzyme involved in purine nucleotide cycling and adenine nucleotide catabolism that catalyzes the conversion of AMP to IMP (Fig. 2). The homozygous variant of the *AMPD2* gene on chromosome 1p13 causes pontocerebellar hypoplasia type 9 (PCH9) [56] and spastic paraplegia-63 (SPG63) [57]. PCH9 is a rare autosomal recessive disorder of purine nucleotide metabolism. The possible pathogenic mechanism of PCH9 is that the homozygous variant of the *AMPD2* gene leads to deficiency of AMPD, which is essential for guanine nucleotide biosynthesis and protein translation. AMPD deficiency leads to increased ATP and decreased GTP levels, resulting in defective initiation of GTP-dependent protein translation. Experiments in AMPD2-deficient mice have found neurodegeneration, and degeneration of CA3 pyramidal neurons in the hippocampus and sparse Pyknotic cells in the cortex and cerebellum may be related to the occurrence of neurological symptoms [56].

The common clinical manifestations of PCH9 include microcephaly, dysphagia, psychomotor retardation, spasticity, seizures, and abnormal brain imaging findings. The neuroimaging MRI of PCH9 shows pontocerebellar hypoplasia, which is characterized by significant atrophy of the "dragonflies" cerebellar hemisphere, relatively sparse vermis, hypoplasia of the corpus callosum, figure-of-eight appearance of the brainstem, and periventricular white matter. Other non-specific manifestations include white matter reduction, brain atrophy, and severe abnormalities of the basal ganglia and thalamus [56, 58].

The diagnosis of PCH9 depends on typical MRI findings, and genetic testing such as linkage analysis, whole exome sequencing, and molecular Sanger sequencing can also help the diagnosis [56, 59]. The EEG of AMPD deficiency may show θ and δ slowing, β activity, and diffuse rhythm slowing [60]. Biochemical measurement of the percentage of adenosine or guanine nucleotide levels in fibroblasts or neural progenitor cells from patients with AMPD deficiency by HPLC suggests that AMPD deficiency causes an increase in ATP and a decrease in GTP [56].

The pathogenesis of AMPD deficiency is mainly neurotoxicity caused by the accumulation of adenosine and GTP-dependent protein translation initiation disorders [56]. Therefore, the treatment of AMPD deficiency can be performed by supplementating the precursors of the purine de novo synthesis pathway. Some studies have shown that the supplementation of AICAr can improve the level of guanine nucleotide defect, reduce the pressure of nerve cells, [56], and thus improve the symptoms AICAr can be converted to AICAR required for de novo purine synthesis under the phosphorylation of adenosine kinase, rescue the de novo purine synthesis disorder caused by AMPD deficiency, and produce IMP. In turn, GMP and GTP can be produced. Increased production of IMP can also be dephosphorylated to form inosine, which activates AMPK, a regulator important for maintaining cellular energy homeostasis [61–65]. It should be noted that supplementation of AMPK activator alone was not effective in the improvement of AMPD deficiency, further indicating that the pathogenic mechanism of AMPD deficiency is guanine nucleotide deficiency caused by purine de novo synthesis disorder and adenosine accumulation mediated neurotoxicity.

### Double-stranded RNA-specific adenosine 2 deaminase deficiency

Double-stranded RNA-specific adenosine deaminase 2 (ADAR2) deficiency is a rare autosomal recessive disorder [66] of nucleic acid metabolism caused by the homozygous or biallelic variant of the *ADARB1* gene on chromosome 21q22 [67]. The *ADARB1* gene encodes the ADAR2 enzyme, which catalyzes A-to-I RNA editing by binding to double-stranded RNA [68]. GRIA2 is a subunit of the ionic α-amino-3- hydroxy-5-methyl-4-isoxazole-propionic acid (AMPA) glutamate receptor. ADAR2 editing converts the glutamine codon (CAG) of GRIA2 to arginine (CIG), making AMPA receptors less calcium-permeable [69]. The increase of calcium permeability of the AMPA receptor leads to calcium influx or abnormal activation of calcium-dependent protease, which may be the pathogenic mechanism of ADAR2 deficiency.

The common clinical manifestations of diseases caused by the *ADARB1* gene variant include neurodevelopmental disorders, hypotonia, microcephaly, seizures, intellectual disability, and limb spasticity [66]. Usually in the first few months of life, patients may have excessive irritability, crying, and feeding difficulties. Seizures usually appear in the first year of life [70], and their seizure types include generalized tonic–clonic seizures, myoclonic seizures, West syndrome manifested infantile spasms, focal seizures, and status epilepticus.

The EEG of ADAR2 deficiency patients showed slow background rhythm and bilateral multifocal epileptic discharges [70]. Most of the neuroimaging findings are non-specific. MRI can show delayed myelination, diffuse brain atrophy with white matter involvement, and agenesis of the corpus callosum [66, 70]. Biochemical examination of patients with *ADARB1* gene variant, such as organic acids and acylcarnitine spectrum, is usually normal. Therefore, the diagnosis of this disease is mainly based on the genetic detection of the *ADARB1* gene variant. Tan et al. reported 4 patients with a biallelic *ADARB1* variant associated with microcephaly, intellectual disability, and seizures in 2020 [66]. Among them, the p. Arg603gln variant leads to a severe reduction in editing activity.

Valproic acid, levetiracetam, phenobarbital, topiramate, and other antiseizure medications are ineffective, and ketogenic diet therapy and vagus nerve stimulation therapy are also ineffective [66]. Seizure control with selective and non-competitive AMPA receptor antagonists, such as perampanel, may be a feasible treatment for ADAR2 deficiency. Early intervention to treat seizures may reduce or delay the progression of brain lesions [70, 71].

### Inosine triphosphate pyrophosphohydrolase deficiency

Inosine triphosphate pyrophosphohydrolase (ITPase) deficiency is a rare autosomal recessive genetic disorder of nucleic acid metabolism. The *ITPA* gene, located on chromosome 20p.13, contains eight exons and encodes ITPase, which plays a critical role in purine metabolism by converting ITP into IMP and pyrophosphate [72]. It protects cells from the accumulation of nucleotides such as ITP, dITP, and xanthine triphosphate (XTP), which can be mistakenly incorporated into RNA and DNA [73]. Variations in the *ITPA* gene impair ITPase activity, leading to deficiency. This deficiency may cause genetic damage to cells, trigger programmed cell death, and interfere with the intracellular signaling pathways related to ATP and GTP [74].

Kevelam et al. first reported seven patients with severe developmental and epileptic encephalopathy in 2015 and identified four novel homozygous variants in the *ITPA* gene [74]. The common clinical manifestations of ITPase deficiency include seizures in the first few months of life, microcephaly, psychomotor retardation, hypotonia, feeding difficulties, and early death in most patients. There are also some other symptoms, including ocular involvement such as cataracts, cardiac involvement such as dilated cardiomyopathy, and appendiceal spasm with hyperreflexia. The seizure types of ITP include febrile seizures, myoclonic seizures, tonic seizures, and generalized tonic–clonic seizures [75].

Severe decrease in ITPase activity and accumulation of ITP were detected in red blood cells, while a severe reduction in ITPase activity without ITP accumulation was observed in fibroblasts. The neuroimaging findings in this disease demonstrate the characteristics of neuronal degeneration, with MRI showing impaired early myelin formation. The characteristic manifestation is obvious T2 hyperintensity and diffusion restriction in the posterior limb of the internal capsule or pyramidal tract [74], as well as involvement of the brainstem tract, primary visual and motor cortex tracts, often accompanied by progressive brain atrophy. Whole exome sequencing and Sanger sequencing can reveal *ITPA* gene variants. Patients with ITPase deficiency usually have no obvious abnormalities in biochemical tests. EEG findings are highly heterogeneous and may show focal, multifocal, or diffuse epileptiform discharges with slow, chaotic background rhythms.

When ITPase is deficient, 6-mercaptopurine metabolism is abnormal. We should pay attention to drug toxicity when using thiopurine and other antineoplastic drugs [76]. During the treatment of hepatitis C with ribavirin, ITPase deficiency may be related to the delayed development of anemia [77, 78]. There is no specific treatment for ITPase deficiency at present. Severe developmental and epileptic encephalopathy caused by ITP should be treated with antiseizure therapy as early as possible. Most of the epilepsy in this disease is refractory epilepsy, and the treatment with one or more antiseizure medications is not effective. The ketogenic diet, as a low-carbohydrate method to reduce neuronal excitability, can be considered as a treatment option for refractory epilepsy caused by ITPase deficiency [75].

### Dihydropyrimidine dehydrogenase deficiency

Dihydropyrimidine dehydrogenase (DPD) deficiency is a rare autosomal recessive disorder of pyrimidine nucleotide metabolism caused by homozygous or compound heterozygous variants in the *DPYD* gene, which is located on chromosome 1p22 and containing 23 exons and is approximately 950 kb in length [79]. The *DPYD* gene encodes DPD, which is involved in the degradation pathway of pyrimidine nucleotides and converts thymine and uracil to their corresponding dihydrogen derivatives [80]. Variants in the *DYPD* gene lead to the deficiency of DPD, which blocks the degradation of thymine and uracil, and its products β-aminosiobutyrate and β-alanine are reduced (Fig. 2). Studies have shown that β-alanine is a GABA reuptake blocker in glial cells [81], and its molecular structure is similar to γ-aminobutyric acid and glycine, which are two major inhibitory neurotransmitters in the nervous system. DPD deficiency leads to decreased production of β-alanine, which may be the cause of neurological symptoms in this disease.

The common clinical manifestations of DPD deficiency include psychomotor retardation, seizures, microcephaly, dystonia, and ocular abnormalities in infancy. In adulthood, because 5-fluorouracil is metabolized by DPD, DPD is the main inhibitor of 5-fluorouracil. Patients with DPD deficiency may suffer from serious life-threatening manifestations such as hyperammonemia encephalopathy after receiving cancer treatment drugs such as 5-fluorouracil and its analogs [82]. Therefore, it is necessary to screen for DPD deficiency before using 5-fluorouracil and its analogs for cancer treatment.

The diagnosis of DPD deficiency can be confirmed by biochemical tests such as gas chromatography-mass spectrometry (GC-MS) to determine the increased levels of thymine and uracil in blood, urine, and cerebrospinal fluid. The DPD enzyme activity in fibroblasts, liver, and blood monocytes and the detection of *DPYD* gene variants are also helpful for the diagnosis of DPD deficiency. Neuroimaging examination of DPD deficiency can show non-specific manifestations such as brain atrophy and white matter abnormalities [83]. The EEG of DPD deficiency can show various types of epileptic discharges [84]. Fleger et al. reported a case of DPD deficiency with mental retardation, psychomotor retardation, microcephaly, and highly active epileptiform discharges on EEG as the main clinical features [85]. In 1990, Brockstedt et al. reported a case of DPD deficiency characterized by seizures, psychomotor retardation, and behavioral abnormalities, with focal sudden sharp waves in the temporo-occipital region and generalized slowing of background activity on EEG [86].

Seizures are a common symptom of DPD deficiency, and antiseizure treatment is also a key part of the treatment of DPD deficiency. Antiseizure medications such as sodium valproate, oxcarbazepine, phenobarbitone, and ethosuximide can control the symptoms of seizures, relieve the epileptiform discharges on EEG, and improve the overall condition and development [83, 84, 86, 87]. Studies have shown that a variety of antiseizure medications can control seizures by enhancing the inhibitory effect of GABA in the nervous system. β-alanine, a metabolite of pyrimidine degradation, as a GABA reuptake blocker, also has antiepileptic effects [88].

### Dihydropyrimidinase deficiency

Dihydropyrimidinase (DHP) deficiency is a rare autosomal recessive disorder affecting pyrimidine nucleotide metabolism. The causative gene, *DPYS*, is located on chromosome 8q22, spanning more than 80 kb in length and comprising 10 exons [89]. The *DPYS* gene encodes DHP, which is involved in the second key step of the pyrimidine degradation metabolic pathway, namely, catalyzing the conversion of dihydrouracil and dihydrothymine into β-ureidopropionic acid and β-ureidoisobutyric acid (Fig. 2). When *DPYS* is varied, DHP deficiency leads to impaired metabolism of pyrimidine degradation, accumulation of dihydrogen derivatives, and decreases in β-alanine and β-aminosiobutyrate, as described above in DPD deficiency. β-alanine is also reduced in DHP deficiency, which may be the cause of neurological disorders. β-aminosiobutyrate, a partial receptor agonist of the major inhibitory neurotransmitter glycine, is significantly reduced in DHP deficiency [90]. It has also been suggested that β-aminosiobutyrate may increase the excretion of leptin and stimulate fatty acid oxidation [91], and leptin has been studied [92] for its neuroprotective, cognitive, and anticonvulsant effects [92–94]. Therefore, a significant reduction in β-aminosiobutyrate in DHP deficiency may also be the cause of neurological symptoms.

The common clinical symptoms of DHP deficiency include neurological symptoms and gastrointestinal symptoms. Neurological symptoms include seizures, mental retardation, growth retardation, microcephaly, and hypotonia, etc. Gastrointestinal symptoms include eating disorders, vomiting, gastroesophageal reflux, and malabsorption with villous atrophy, etc [95]. Nakajima et al. reported four patients with DHP deficiency in 2017 [96]. One of the patients presented with infantile spasms and head nodding at the age of 4 months, and a head MRI showed decreased white matter and brain atrophy.

Biochemical examination of patients with DHP deficiency can show increased dihydrouracil and dihydrothymine in blood, urine, and cerebrospinal fluid, and decreased β-alanine and β-aminosiobutyrate. The enzymatic examination of DHP can only rely on liver biopsy to determine its enzyme activity. This is because unlike DPD, DHP exists in the liver and kidney. *DPYS* gene variant detection can also help to diagnose the disease. Neuroimaging studies of DHP deficiency can show secondary delayed myelination, progressive neuronal atrophy, white matter reduction, and brain atrophy [95–98].

There is no specific treatment for this disease at present, and most of them are symptomatic treatments, which is similar to the antiepileptic treatment for DPD deficiency. Attention should be paid to the existence of pyrimidine degradation and metabolism enzyme deficiency when using antineoplastic drugs such as 5-fluorouracil, to avoid more serious disorders.

### β-ureidopropionase deficiency

β-Ureidopropionase (βUP) deficiency is a rare autosomal recessive disorder of pyrimidine nucleotide metabolism caused by homozygous or compound heterozygous variants in the *UPB1* gene on chromosome 22q11. The *UPB1* gene encodes β-ureidopropionase, which is involved in the final step of the pyrimidine nucleotide degradation pathway, namely the conversion of N-carbamoyl-β-alanine (β-ureidopropionic acid) and N-carbamoyl-β-aminoisobutyric acid (β-ureoisobutyric acid) to β-alanine and β-aminosiobutyrate (Fig. 2). When the *UPB1* gene is varied, it leads to the deficiency of β-ureidopropionase, which in turn causes a series of clinical manifestations. Some studies have suggested that changes in β-aminoisobutyric acid homeostasis and/or increased oxidative stress may lead to clinical manifestations such as epilepsy in β-ureypropionate deficiency [4].

Similar to DPD deficiency and DHP deficiency, patients with βUP deficiency present with neurological symptoms, such as hypotonia, intellectual disability, severe developmental delay, and seizures, as well as scoliosis and congenital abnormalities of the urogenital system and colorectal system [99]. Van Kuilenburg et al. reported four patients with βUP deficiency [4]. One of them presented with febrile seizures, bradykinesia, hypsarrhythmia on EEG, and delayed myelination on head MRI at the age of 8 months. After 1 year of treatment with vigabatrin, the EEG symptoms were relieved. At the age of 3 years, the child presented with psychomotor retardation, severe intellectual disability, and speech disorder. Lee et al. reported a case of βUP deficiency with generalized tonic–clonic seizures as the initial presentation in 2014 [100]. The patient presented with non-specific EEG and neuroimaging findings. The patient presented with generalized tonic–clonic seizures at 3 months of age, and the seizures stopped after the administration of phenyobatoid. The patient developed recurrent seizures, including generalized status epilepticus and refractory seizures, which were unresponsive to various antiepileptic drugs and ketogenic diet therapy. At the age of 8 years, she developed generalized hypotonia and was unable to speak or sit alone. At the age of 9 years and 1 month, a restricted purine and pyrimidine diet was used to reduce the frequency of fall attacks and generalized tonic–clonic seizures. At the same time, a variety of antiseizure medications were used, including valproic acid, zonisamide, rufilamide, and clobazam.

Biochemical tests for βUP deficiency showed normal or moderately elevated levels of dihydrouracil and dihydrothymine, and significantly elevated levels of β-ureidopropionic acid and β-ureidoisobutyric acid in the blood, urine, and cerebrospinal fluid [101]. As in DHP deficiency, enzymatic testing of β- ureidopropionic acid requires determination of its enzyme activity by liver biopsy [102]. The neuroimaging examination of this disease, MRI, shows nonspecific manifestations such as delayed myelination and brain atrophy. The diagnosis of the disease requires biochemical examination, enzyme examination, and genetic testing.

There is no specific treatment for βUP deficiency at present. β-alanine, β-aminosiobutyrate supplements, and βUP supplementation to help β-ureidopropionic acid and β-ureidoisobutyric acid metabolism may be applied methods, but studies on the application of β-alanine have not shown clinical improvement results [103]. However, the application of β-alanine has not shown clinical improvement. The other two treatment methods have not been reported in the treatment of this disease. Restriction of the pyrimidine diet may be a more effective method, which has been reported to reduce the frequency of seizures [100]. The antiseizure medication therapy is also a symptomatic treatment for this disease (Table 1).

**Table 1:** The pathogenic genes, inheritance, seizure types, EEG, and treatment of disorders of nucleic acid/nucleotide metabolism associated with epilepsy

Syndrome	Pathogenic gene	Inheritance	Types of seizures	EEG	Treatment
Adenosine succinate lyase deficiency	*ADSL*	AR	Myoclonic seizures, focal seizures, GTCS, status epilepticus, infantile spasms, etc	Extensive spikes and waves, burst-suppression manifestations, etc	D-ribose, uridine, ASM, ketogenic diet
Lesch-Nyhan syndrome	*HPRT*	XLR		Nonspecific slowing or disorder	SAMe, risperidone, baclofen, benzodiazepines, gabapentin
ATIC deficiency	*ATIC*	AR	Epileptic spasms, generalized tonic seizures, focal seizures with disturbance of consciousness, etc		ASM
Adenosine monophosphate deaminase deficiency	*AMPD2*	AR		θ and δ slowing, β activity and diffuse rhythm slowing	Supplementing the precursors of the purine de novo synthesis pathway
Double-stranded RNA-specific adenosine deaminase 2 deficiency	*ADARB1*	AR	GTCS, myoclonic seizures, West syndrome manifested infantile spasms, focal seizures, and status epilepticus	Slow background rhythm and bilateral multifocal epileptic discharges	ASM (VPA, LEV, PB, PER)
Inosine triphosphate pyrophosphohydrolase deficiency	*ITPA*	AR	Febrile seizures, myoclonic seizures, tonic seizures, and generalized tonic–clonic seizures	Focal, multifocal, or diffuse epileptiform discharges with slow, chaotic background rhythms	ASM, ketogenic diet
Dihydropyrimidine dehydrogenase deficiency	*DPYD*	AR		Sharp waves in the temporo-occipital region and generalized slowing of background activity	ASM (VPA, OXC, PB)
Dihydropyrimidinase deficiency	*DPYS*	AR	Infantile spasms		ASM
β-ureidopropionase deficiency	*UPB1*	AR	GTCS, febrile seizures, etc	Hypsarrhythmia	ASM (VPA, ZNS), β-alanine, β-aminosiobutyrate supplements, and βUP supplementation

*Abbreviations: AR* Autosomal recessive, *ASM* Antiseizure medication, *ATIC* Amino imidazole carboxamide ribosidetransformylase/I inosine monophosphorus cyclohydrolase, *EEG* Electroencephalogram, *GTCS* Generalized tonic–clonic seizure, *LEV* Levetiracetam, *OXC* Oxcarbazepine, *PB* Phenobarbital, *PER* Perampanel, *SAMe* S-adenosine-L-methionine, *VPA* Valproic Acid, *XLR* X-linked recessive, *ZNS* Zonisamide, *βUP* β-ureidopropionase

## Conclusions

Nucleic acid/nucleotide metabolism disorders include a range of rare clinical syndromes caused by disruption of the activity of enzymes necessary for nucleic acid synthesis in the human body or by mutations in the genes that code for them. These syndromes present different clinical manifestations. This review article provides an overview of diseases related to nucleic acid/nucleotide metabolism and epilepsy, including seizure types such as myoclonic seizures, focal seizures, generalized tonic–clonic seizures, febrile seizures, and West syndrome. There is a lack of large-scale clinical trials and a limited understanding of its molecular mechanisms. Most of this type of epilepsy is drug-resistant or refractory epilepsy. This article reviews several possible antiepileptic treatments, such as a low carbohydrate ketogenic diet reducing neuronal excitability. It can be used in adenylate succinate lyase deficiency and inosine triphosphatase deficiency. Gabapentin, baclofen, and benzodiazepines may be used for Lesch-Nyhan syndrome. Perampanel is an effective treatment for epilepsy with double-stranded RNA-specific adenosine deaminase deficiency. Antiseizure medications that enhance GABA inhibition in the nervous system can be used to control seizures in patients with DPD deficiency. A restricted pyrimidine diet may be a viable approach for antiepileptic therapy in patients with β-propionate uracil deficiency. The use of certain antiseizure medications in the treatment of seizures caused by nucleic acid/nucleotide metabolism disorders summarized above is mainly based on the clinical manifestations of the type of seizures to select a more appropriate antiseizure medication. At present, the mechanism of seizures caused by nucleic acid/nucleotide metabolism disorders is not clear. There is also no clear interaction between its pathogenesis and the action mechanism of antiepileptic drugs.

We anticipate further exploration of the molecular mechanisms underlying this condition as more research emerges on potential therapeutic approaches, such as gene therapy. In future clinical work, we will also strive to collect clinical cases, and improve the theoretical framework and practical application.

## Data Availability

Not applicable.
